# Technical data for concentrated solar power plants in operation, under construction and in project

**DOI:** 10.1016/j.dib.2017.06.030

**Published:** 2017-06-23

**Authors:** Ugo Pelay, Lingai Luo, Yilin Fan, Driss Stitou, Mark Rood

**Affiliations:** a Laboratoire de Thermique et Energie de Nantes, UMR CNRS 6607, Polytech׳ Nantes – Université de Nantes, La Chantrerie, Rue Christian Pauc, BP 50609, 44306 Nantes Cedex 03, France; b Laboratoire PROcédés, Matériaux et Energie Solaire (PROMES), UPR CNRS 8521, Rambla de la Thermodynamique, Tecnosud, 66100 Perpignan, France; c Department of Civil and Environmental Engineering, University of Illinois at Urbana-Champaign, 205N. Mathews Avenue, IL 61801, USA

## Abstract

This article presents technical data for concentrated solar power (CSP) plants in operation, under construction and in project all over the world in the form of tables. These tables provide information about plants (e.g., name of the CSP plant, country of construction, owner of the plant, aim of the plant) and their technical characteristics (e.g., CSP technology, solar power, area of the plant, presence and type of hybridization system, electricity cost, presence and type of TES, power cycle fluid, heat transfer fluid, operating temperature, operating pressure, type of turbine, type and duration of storage, etc.). Further interpretation of the data and discussions on the current state-of-the-art and future trends of CSP can be found in the associated research article (Pelay et al., 2017) [1].

**Specifications Table**

**Table:** 

Subject area	*Energy*
More specific subject area	*Solar Energy*
Type of data	*Table*
How data was acquired	*Survey*
Data format	*Raw*
Experimental factors	*Not applicable*
Experimental features	*Not applicable*
Data source location	*Not applicable*
Data accessibility	*Data are with this article*

**Value of the data**

• A detailed database for the technical data of CSP plants all over the world
• Make possible an analysis of actual state-of-the-art of CSP plants
• Make possible an analysis of future trends on CSP plants

## 1 Data

Technical data for concentrated solar power (CSP) plants in operation, under construction and in project all over the world in the form of tables. These tables provide information about plants (e.g., name of the CSP plant, country of construction, owner of the plant, aim of the plant) and their technical characteristics (e.g., CSP technology, solar power, area of the plant, presence and type of hybridization system, electricity cost, presence and type of TES, power cycle fluid, heat transfer fluid, operating temperature, operating pressure, type of turbine, type and duration of storage, etc.). Further interpretation of the data and discussions on the current state-of-the-art and future trends of CSP can be found in the associated research article [1].

## 2 Experimental design, materials and methods

First, a large part of the data have been collected from the websites “cspworld.org” and “globalenergyobservatory.org”. Then a detailed research has also been made for each identified CSP plant on its proper website or related documents so as to find additional information.
