# Supplementary material for: Technical data for concentrated solar power plants in operation, under construction and in project
Source: Data Brief. 2017 Jun 23;13:597–9. doi: 10.1016/j.dib.2017.06.030 (PMC5499030; doi:10.1016/j.dib.2017.06.030)
Supplement: Supplementary file 2 — Supplementary material [file mmc2.docx]

**Table 1: Technical data for concentrated solar power plants in operation [1], [2]**

| **Project** | **Country** | **Owner** | **CSP Technology** | **Solar power (MWel)** | **Generation (GW.h /year)** | **Purpose of the plant** | **Hybridization** | **Area of the plant (hectare)** | **Electricity cost (€/kW.h)** | **Type of power cycle - fluid** | **Heat transfer fluid** | **Operating tempe-rature (°C)** | **Operating pressure (bar)** | **Type of Turbin** | **Type of cooling** | **mirror's area (m^2)** | **Type of storage** | **Mean of storage** | **Storage capacity (h)** | **Start date** |
| --- | --- | --- | --- | --- | --- | --- | --- | --- | --- | --- | --- | --- | --- | --- | --- | --- | --- | --- | --- | --- |
| Cooma tower | Australia | Solastor | SPT | n.a. | n.a. | Demonstration | n.a. | n.a. | n.a. | n.a. | n.a. | n.a. | n.a. | n.a. | n.a. | n.a. | n.a. | n.a. | n.a. | n.a. |
| E Cube Energy Dish pilot plant | China | E cube Energy | PDC | 1 | 1.5 | Demonstration | No backup | 4 | n.a. | n.a. | n.a. | n.a. | n.a. | n.a. | n.a. | n.a. | n.a. | n.a. | n.a. | n.a. |
| IIT Madras R&D facilities | India | n.a. | PTC | n.a. | n.a. | R&D | n.a. | n.a. | n.a. | n.a. | n.a. | n.a. | n.a. | n.a. | n.a. | n.a. | n.a. | n.a. | n.a. | n.a. |
| The Ben-Gurion National Solar Energy Center | Israel | n.a. | PTC | n.a. | n.a. | R&D | n.a. | n.a. | n.a. | n.a. | n.a. | n.a. | n.a. | n.a. | n.a. | n.a. | n.a. | n.a. | n.a. | n.a. |
| Dhursar | India | Reliance Power | LFR | 125 | 280 | Commercial | n.a. | 340 | 0.15 | Rankine - Steam | n.a. | n.a. | n.a. | n.a. | Wet (tower) | n.a. | No storage | No storage | No storage | 2014 |
| Medecine Hat ISCC | Canada | n.a. | PTC | 1.1 | n.a. | Commercial | n.a. | n.a. | n.a. | n.a. | n.a. | n.a. | n.a. | n.a. | n.a. | 5248 | n.a. | n.a. | n.a. | 2014 |
| MTN CSP cooling plant | South Africa | n.a. | LFR | 0.33 | n.a. | Commercial | n.a. | n.a. | n.a. | n.a. | n.a. | n.a. | n.a. | n.a. | n.a. | n.a. | n.a. | n.a. | n.a. | 2014 |
| Airlight Energy Baha Plant [3] | Morocco | Cimar, Italcementi Group | PTC | 3 | 2.4 | Demonstration | No backup | 24 | n.a. | Rankine - Organic | Air | 650 | n.a. | Turboden ORC Turbine | n.a. | 6160 | Sensible | Stone | 12 | 2013 |
| Archimede-Chiyoda Molten Salt Test Loop | Italia | Archimede/ Chiyoda | PTC | 0.35 | 0.28 | R&D | n.a. | n.a. | n.a. | Rankine - Steam | n.a. | 550 | n.a. | Siemens | n.a. | 3398 | Sensible | Molten salts (2 direct tanks) | 5 | 2013 |
| Arenales | Spain | OHL RREEF STEAG | PTC | 50 | 170 | Commercial | Solar/Natural Gas(Evaporator HTF-15% max) | 220 | 0.27 | Rankine - Steam | Diphyl | 393 | n.a. | GE | Wet | 510120 | Sensible | Molten salts (2 Indirect tanks) | 7 | 2013 |
| BBEnergy Eskom 150 kW Fresnel pilot plant | South Africa | Eskom | LFR | 0.15 | 0.5 | R&D | n.a. | n.a. | n.a. | n.a. | Water | 250 | n.a. | n.a. | n.a. | n.a. | n.a. | n.a. | n.a. | 2013 |
| Caceres | Spain | Cobra | PTC | 50 | 170 | Commercial | Solar/Natural Gas(Evaporator HTF-15% max) | 200 | 0.27 | Rankine - Steam | Biphenyl / Diphenyl Oxide | 393 | 100 | Siemens SST700 | Wet (tower) | 510120 | Sensible | Molten salts (2 Indirect tanks) | 7,5 | 2013 |
| Casablanca | Spain | Cobra | PTC | 50 | 170 | Commercial | Solar(1-2% of the production)/ Charbon | 200 | 0.27 | Rankine - Steam | Biphenyl / Diphenyl Oxide | 390 | 100 | Siemens SST700 | Wet (tower) | 510120 | Sensible | Molten salts (2 Indirect tanks) | 7,5 | 2013 |
| Enerstar Villena | Spain | FCC Energia | PTC | 50 | 100 | Commercial | Solar/Natural Gas(Evaporator HTF-15% max) | 214 | 0.27 | Rankine - Steam | Thermal oil | 393 | 100 | MAN Diesel & Turbo - dual casing reheat turbine | Wet (tower) | 339506 | No storage | No storage | No storage | 2013 |
| Godawari Solar Project | India | Hira Group | PTC | 50 | 118 | Commercial | No backup | 150 | 0.16 | Rankine - Steam | Dowtherm A (Biphenyl / Diphenyl Oxide) | 390 | n.a. | Siemens SST700 | Wet | 394000 | No storage | No storage | No storage | 2013 |
| Ivanpah Solar Electric Generating System | USA | BirgthSource Energy, Google, NGR Energy | SPT | 392 | 1079 | Commercial | Solar/ Natural Gas | 1416 | n.a. | Rankine - Steam | Water | 565 | 160 | Siemens SST900 | Dry | 3E+06 | No storage | No storage | No storage | 2013 |
| KGDS Narippaiyur desalination CSP plant | India | KGDS Renewable Energy | LFR | n.a. | n.a. | Commercial | No backup | n.a. | n.a. | n.a. | n.a. | n.a. | n.a. | n.a. | n.a. | 1400 | No storage | No storage | No storage | 2013 |
| Shams 1 | United Arab Emirates | Abengoa Solar, Masdar, Toral | PTC | 100 | 210 | Commercial | Solar /Natural Gas (Evaporator) | 250 | n.a. | Rankine - Steam | Therminol VP-1 (Biphenyl / Diphenyl Oxide) | 393 | n.a. | MAN Diesel & Turbo | Dry | 627840 | No storage | No storage | No storage | 2013 |
| Solaben 1 | Spain | Abengoa Solar | PTC | 50 | 100 | Commercial | Solar /Natural Gas (Evaporator) | 70 | 0.27 | Rankine - Steam | Thermal oil | 393 | 100 | n.a. | Wet (tower) | 300000 | No storage | No storage | No storage | 2013 |
| Solaben 6 | Spain | Abengoa Solar | PTC | 50 | 100 | Commercial | Solar /Natural Gas (Evaporator) | 110 | 0.27 | Rankine - Steam | Thermal oil | 393 | 100 | n.a. | Wet (tower) | 300000 | No storage | No storage | No storage | 2013 |
| Solana Generating Station | USA | Abengoa Solar | PTC | 280 | 944 | Commercial | Solar /Natural Gas (Evaporator) | 1257 | n.a. | Rankine - Steam | Therminol VP-1 (Biphenyl / Diphenyl Oxide) | 393 | 100 | n.a. | Wet | 2E+06 | Sensible | Molten salts (2 Indirect tanks) | 6 | 2013 |
| Solugas | Spain | Abengoa Solar | SPT | 4.6 | n.a. | R&D | n.a. | n.a. | n.a. | Brayton | Air | 800 | n.a. | Turbomach Mercury 50TM | n.a. | n.a. | No storage | No storage | No storage | 2013 |
| Termesol 1 | Spain | NextEra | PTC | 50 | 170 | Commercial | Solar /Natural Gas (Evaporator) | 200 | 0.27 | Rankine - Steam | Biphenyl / Diphenyl Oxide | 393 | n.a. | n.a. | Wet | 523200 | Sensible | Molten salts (2 Indirect tanks) | 9 | 2013 |
| Termesol 2 | Spain | NextEra | PTC | 50 | 170 | Commercial | Solar /Natural Gas (Evaporator) | 200 | 0.27 | Rankine - Steam | Biphenyl / Diphenyl Oxide | 393 | n.a. | n.a. | Wet | 523200 | Sensible | Molten salts (2 Indirect tanks) | 9 | 2013 |
| AORA Solar Tulip Tower-Almeria | Spain | AORA | SPT | 0.1 | n.a. | R&D | n.a. | n.a. | n.a. | n.a. | n.a. | n.a. | n.a. | n.a. | n.a. | n.a. | n.a. | n.a. | n.a. | 2012 |
| Aste 1A | Spain | Aries/Eiser/ Elecnor | PTC | 50 | 170 | Commercial | Solar/Natural Gas(Evaporator HTF-15% max) | 180 | 0.27 | Rankine - Steam | Dowtherm A (Biphenyl / Diphenyl Oxide) | 393 | 100 | n.a. | Wet (tower) | 510120 | Sensible | Molten salts (2 Indirect tanks) | 8 | 2012 |
| Aste 1B | Spain | Aries/Eiser/ Elecnor | PTC | 50 | 170 | Commercial | Solar/Natural Gas(Evaporator HTF-15% max) | 180 | 0.27 | Rankine - Steam | Dowtherm A (Biphenyl / Diphenyl Oxide) | 393 | 100 | n.a. | Wet (tower) | 510120 | Sensible | Molten salts (2 Indirect tanks) | 8 | 2012 |
| Astexol II | Spain | Aries/Eiser/ Elecnor | PTC | 50 | 170 | Commercial | Solar/Natural Gas(Evaporator HTF-15% max) | 160 | n.a. | Rankine - Steam | Thermal oil | 393 | 100 | GE | Wet (tower) | 510120 | Sensible | Molten salts (2 Indirect tanks) | 7,5 | 2012 |
| Augustin Fresnel | France | Solar Euromed | LFR | 0.25 | n.a. | Demonstration | No backup | 1 | n.a. | Rankine - Steam | Water | 300 | n.a. | n.a. | Dry | 400 | Other | Ruths tanks | 0,25 | 2012 |
| Borges Termosolar | Spain | Abantia, Comsa Emte | PTC | 22.5 | 98 | Commercial | Solar/Biomass/ Natural Gas | n.a. | 0.27 | Rankine - Steam | Dowtherm A (Biphenyl / Diphenyl Oxide) | 393 | n.a. | MAN Diesel & Turbo MARC 6-R05 | Wet | 183120 | No storage | No storage | No storage | 2012 |
| CTAER variable geometry solar test facility | Spain | n.a. | SPT | 8 | n.a. | R&D | No backup | n.a. | n.a. | n.a. | n.a. | n.a. | n.a. | n.a. | n.a. | 1560 | n.a. | n.a. | n.a. | 2012 |
| Dahan Power Plant | China | Institute of electrical engineeringof chenese academy of sciences | SPT | 1 | n.a. | Demonstration | Solar/Oil | 5 | n.a. | Rankine - Steam | Water/Steam | 400 | n.a. | n.a. | Wet | 10000 | Sensible | Oil / Steam | 1 | 2012 |
| Exresol-3 | Spain | Cobra, GE, KGAL | PTC | 50 | 170 | Commercial | Solar/Natural Gas(Evaporator HTF-15% max) | 200 | 0.27 | Rankine - Steam | Diphenyl/Diphenyl Oxide | 393 | 100 | Siemens SST700 | Wet (tower) | 510120 | Sensible | Molten salts (2 Indirect tanks) | 7,5 | 2012 |
| Greenway | Turkey | Greenway CSP | SPT | 1 | n.a. | Demonstration | n.a. | n.a. | n.a. | Rankine - Steam | Water | n.a. | 55 | n.a. | n.a. | n.a. | n.a. | Molten salts (3 phases tank, natural circulation) | n.a. | 2012 |
| Guzmán | Spain | FCC Mitsui | PTC | 50 | 100 | Commercial | Solar/Natural Gas(Evaporator HTF-15% max) | 200 | 0.27 | Rankine - Steam | Dowtherm A (Biphenyl / Diphenyl Oxide) | 393 | 100 | MAN Diesel & Turbo - dual casing reheat turbine | Wet (tower) | 310000 | No storage | No storage | No storage | 2012 |
| Helioenergy 2 | Spain | Abengoa Solar EON | PTC | 50 | 100 | Commercial | Solar/Natural Gas(Evaporator HTF-15% max) | 110 | 0.27 | Rankine - Steam | Therminol VP-1 (Biphenyl / Diphenyl Oxide) | 393 | 100 | Siemens SST700 | Wet (tower) | 300000 | No storage | No storage | No storage | 2012 |
| Helios I | Spain | Abengoa Solar | PTC | 50 | 100 | Commercial | Solar/Natural Gas(Evaporator HTF-15% max) | 100 | 0.27 | Rankine - Steam | Therminol VP-1 (Biphenyl / Diphenyl Oxide) | 393 | 100 | Siemens SST700 | Wet (tower) | 300000 | No storage | No storage | No storage | 2012 |
| Helios II | Spain | Abengoa Solar | PTC | 50 | 100 | Commercial | Solar/Natural Gas(Evaporator HTF-15% max) | 100 | 0.27 | Rankine - Steam | Therminol VP-1 (Biphenyl / Diphenyl Oxide) | 393 | 100 | Siemens SST700 | Wet (tower) | 300000 | No storage | No storage | No storage | 2012 |
| La Africana | Spain | Grupo Ortiz, Magtel TSK | PTC | 50 | 170 | Commercial | Solar/Natural Gas(Evaporator HTF-15% max) | 252 | 0.27 | Rankine - Steam | Diphenyl/Biphenyl Oxide | 393 | n.a. | n.a. | Wet | 550000 | Sensible | Molten salts (2 Indirect tanks) | 7,5 | 2012 |
| Liddell Power Station | Australia | Macquarie Generation | LFR | 6 | n.a. | Commercial | n.a. | 270 | n.a. | n.a. | Water/Steam | n.a. | n.a. | n.a. | n.a. | 18490 | n.a. | n.a. | n.a. | 2012 |
| Minera El Tesoro Termosolar | Chile | Minera El Tesoro | PTC | 7 th | 24 | Commercial | n.a. | 7 | n.a. | n.a. | n.a. | n.a. | n.a. | n.a. | n.a. | 16742 | n.a. | n.a. | n.a. | 2012 |
| Moron | Spain | Ibereolica | PTC | 50 | 100 | Commercial | Solar /Natural Gas (Evaporator) | 160 | n.a. | n.a. | Dowtherm A (Biphenyl / Diphenyl Oxide) | 393 | n.a. | Siemens SST700 | n.a. | 400000 | No storage | No storage | No storage | 2012 |
| Nationnal Solar Thermal Power Facility | India | IIT Bombay, Ministry of new and renewable energy of India | PTC | 1 | n.a. | R&D | n.a. | n.a. | n.a. | n.a. | Therminol VP-1 (Biphenyl / Diphenyl Oxide) | n.a. | n.a. | n.a. | n.a. | n.a. | n.a. | n.a. | n.a. | 2012 |
| Olivenza 1 | Spain | Ibereolica | PTC | 50 | 100 | Commercial | Solar /Natural Gas (apport evapo) | n.a. | 0.27 | Rankine - Steam | Dowtherm A (Biphenyl / Diphenyl Oxide) | 393 | n.a. | Siemens SST700 | n.a. | 400000 | No storage | No storage | No storage | 2012 |
| Orellana | Spain | Acciona Energia | PTC | 50 | 100 | Commercial | n.a. | n.a. | 0.27 | Rankine - Steam | Thermal oil | 393 | n.a. | n.a. | Wet | 405000 | No storage | No storage | No storage | 2012 |
| Petroleum Development Oman EOR plant | Oman | Petroleum Development Oman | PTC | 7 th | n.a. | Commercial | n.a. | 1,7 | n.a. | n.a. | Water | n.a. | 100 | n.a. | n.a. | n.a. | n.a. | n.a. | n.a. | 2012 |
| Puerto Errado 2 Thermosolar Power Plant | Spain | Novatec Solar | LFR | 30 | 50 | Commercial | n.a. | n.a. | 0.27 | n.a. | Steam | 270 | 55 | Thermodyn SAS | Dry | 302000 | Sensible | 1 tank thermocline | 1,5 | 2012 |
| Solaben 2 | Spain | Abengoa Solar, Itochu | PTC | 50 | 100 | Commercial | Solar /Natural Gas (Evaporator) | 110 | 0.27 | Rankine - Steam | Thermal oil | 393 | 100 | n.a. | Wet (tower) | 300000 | No storage | No storage | No storage | 2012 |
| Solaben 3 | Spain | Abengoa Solar, Itochu | PTC | 50 | 100 | Commercial | Solar /Natural Gas (Evaporator) | 110 | 0.27 | Rankine - Steam | Thermal oil | 393 | 100 | n.a. | Wet (tower) | 300000 | No storage | No storage | No storage | 2012 |
| Solacor 1 | Spain | Abengoa Solar, JGC | PTC | 50 | 100 | Commercial | Solar /Natural Gas (Evaporator) | 110 | 0.27 | Rankine - Steam | Thermal oil | 393 | 100 | n.a. | Wet (tower) | 300000 | No storage | No storage | No storage | 2012 |
| Solacor 2 | Spain | Abengoa Solar, JGC | PTC | 50 | 100 | Commercial | Solar /Natural Gas (Evaporator) | 110 | 0.27 | Rankine - Steam | Thermal oil | 393 | 100 | n.a. | Wet (tower) | 300000 | No storage | No storage | No storage | 2012 |
| Solnova 1 | Spain | Abengoa Solar | PTC | 50 | 100 | Commercial | Solar /Natural Gas (Evaporator) | 110 | 0.27 | Rankine - Steam | Thermal oil | 393 | 100 | Siemens SST700 | Wet (tower) | 300000 | No storage | No storage | No storage | 2012 |
| Thai Solar Energy 1 | Thailand | Thai Solar Energy | PTC | 5 | n.a. | Commercial | No backup | 110 | 0.28 | n.a. | Water/Steam | 340 | 30 | MAN Diesel & Turbo MARC 2-C04 | Wet (tower) | n.a. | No storage | No storage | No storage | 2012 |
| Xinjian Turpan | China | Guodian | PTC | 0.18 | n.a. | R&D | n.a. | n.a. | n.a. | n.a. | n.a. | n.a. | n.a. | n.a. | n.a. | n.a. | n.a. | n.a. | n.a. | 2012 |
| Yanqing Solar Thermal Power (Dahan Tower Plant) | China | IEE-CAS | SPT | 1 | 1.95 | R&D | Solar/Oil | n.a. | n.a. | Rankine - Steam | Water | 400 | n.a. | Hangzhou Steam Turbine Company | Wet | 10000 | n.a. | 2 stages ( Staturated steam/ Oil) | 1 | 2012 |
| ACME Solar Tower | India | ACME | SPT | 2.5 | n.a. | Demonstration | n.a. | 5 | n.a. | Rankine - Steam | Water/Steam | 420 | 60 | MaxWatt | Wet (tower) | 16222 | No storage | No storage | No storage | 2011 |
| Aïn Beni Mathar ISCC | Morocco | Office Nationale de l'Electricité | PTC | 20 | 55 | Commercial | Solar /Natural Gas | n.a. | n.a. | Rankine - Steam | Therminol VP-1 (Biphenyl / Diphenyl Oxide) | 393 | n.a. | CCI | Dry | 183120 | No storage | No storage | No storage | 2011 |
| Andasol-3 | Spain | Ferrostaal RWE Solar Milenium | PTC | 50 | 170 | Commercial | Solar/Natural Gas(Evaporator HTF-15% max) | 200 | 0.27 | Rankine - Steam | Thermal oil | 393 | 100 | MAN Diesel & Turbo - Dual Casing reheat turbin | Wet (tower) | 510000 | Sensible | Molten salts (2 direct tanks) | 7,5 | 2011 |
| Arcosol 50 | Spain | Torresol Energy | PTC | 50 | 170 | Commercial | Solar/Natural Gas(Evaporator HTF-15% max) | 230 | n.a. | Rankine - Steam | Diphenyl/Diphenyl Oxide | 393 | 100 | n.a. | Wet | 510120 | Sensible | Molten salts (2 direct tanks) | 7,5 | 2011 |
| Berry Petroleum EOR plant | USA | Berry Petroleum | PTC | 7 | n.a. | Demonstration | n.a. | n.a. | n.a. | n.a. | n.a. | n.a. | n.a. | n.a. | n.a. | n.a. | n.a. | n.a. | n.a. | 2011 |
| Coalinga | USA | Chevron | SPT | 29 | n.a. | Commercial (Steam generation) | n.a. | 40 | n.a. | n.a. | n.a. | n.a. | n.a. | n.a. | n.a. | 194000 | n.a. | n.a. | n.a. | 2011 |
| CSIRO Brayton | Australia | CSIRO | SPT | 1 | n.a. | n.a. | n.a. | n.a. | n.a. | n.a. | Air | n.a. | n.a. | n.a. | n.a. | n.a. | No storage | No storage | No storage | 2011 |
| Daegu Solar Power Tower | South Corea | Daesung Energ | SPT | 0.2 | n.a. | R&D | No backup | 2 | n.a. | n.a. | n.a. | n.a. | n.a. | n.a. | n.a. | n.a. | n.a. | n.a. | n.a. | 2011 |
| Eureka GDV direct steam generation | Spain | Abengoa Solar | PTC | n.a. | n.a. | R&D | n.a. | n.a. | n.a. | n.a. | Water | n.a. | n.a. | n.a. | n.a. | n.a. | n.a. | n.a. | n.a. | 2011 |
| Gemasolar Thermosolar Plant | Spain | Torresol Energy | SPT | 19.9 | 110 | Commercial | Solar/Natural Gas(Evaporator HTF-15% max) | 195 | 0.27 | Rankine - Steam | Molten salts | 565 | n.a. | Siemens SST600 | Wet | 304750 | Sensible | Molten salts (2 direct tanks) | 15 | 2011 |
| Hassi R'mel ISCC | Algeria | Abengoa, NEAL, Sonatrach | PTC | 25 | n.a. | Commercial | No backup | n.a. | n.a. | Rankine - Steam | Therminol VP-1 (Biphenyl / Diphenyl Oxide) | 393 | n.a. | Siemens SST900 | Dry | 183860 | No storage | No storage | No storage | 2011 |
| Helioenergy 1 | Spain | Abengoa Solar EON | PTC | 50 | 100 | Commercial | Solar/Natural Gas(Evaporator HTF-15% max) | 110 | 0.27 | Rankine - Steam | Therminol VP-1 (Biphenyl / Diphenyl Oxide) | 393 | 100 | Siemens SST700 | Wet (tower) | 300000 | No storage | No storage | No storage | 2011 |
| ISCC Kuraymat | Egypt | NREA | PTC | 20 | 34 | Commercial | No backup | n.a. | n.a. | Rankine - Steam | Therminol VP-1 (Biphenyl / Diphenyl Oxide) | 393 | n.a. | Siemens SST900 | Wet (tower) | 130800 | No storage | No storage | No storage | 2011 |
| La Dehesa | Spain | Renovables SAMCA | PTC | 50 | 170 | Commercial | Solar/Natural Gas(Evaporator HTF-15% max) | 200 | 0.27 | Rankine - Steam | Diphenyl/Biphenyl Oxide | 393 | 100 | Siemens SST700 | Wet | 552750 | Sensible | Molten salts (2 Indirect tanks) | 7,5 | 2011 |
| Lake Cargelligo | Australia | Graphite Energy | SPT | 3.5 | n.a. | Demonstration | No backup | n.a. | n.a. | Rankine - Steam | Water/Steam | 500 | n.a. | n.a. | n.a. | 6080 | Sensible | Graphit blocs | n.a. | 2011 |
| Lebrija 1 | Spain | Siemens, Valoriza | PTC | 50 | 100 | Commercial | Solar/Natural Gas(Evaporator HTF-15% max) | n.a. | 0.27 | Rankine - Steam | Therminol VP-1 (Biphenyl / Diphenyl Oxide) | 395 | n.a. | Siemens SST700 | Wet | 412020 | No storage | No storage | No storage | 2011 |
| Manchasol-1 | Spain | Cobra | PTC | 50 | 170 | Commercial | Solar/Natural Gas(Evaporator HTF-15% max) | n.a. | 0.27 | Rankine - Steam | Dowtherm A (Biphenyl / Diphenyl Oxide) | 393 | n.a. | Siemens SST700 | Wet | 510000 | Sensible | Molten salts (2 Indirect tanks) | 7,5 | 2011 |
| Manchasol-2 | Spain | Cobra | PTC | 50 | 170 | Commercial | Solar/Natural Gas(Evaporator HTF-15% max) | n.a. | 0.27 | Rankine - Steam | Dowtherm A (Biphenyl / Diphenyl Oxide) | 393 | n.a. | Siemens SST700 | Wet | 510000 | Sensible | Molten salts (2 Indirect tanks) | 7,5 | 2011 |
| MicroCSP Process Heat at Tokyo | Japan | n.a. | PTC | 0.1 th | n.a. | Commercial | n.a. | n.a. | n.a. | n.a. | Xceltherm 600 (Thermal oil) | 176 | n.a. | n.a. | n.a. | n.a. | Sensible | Storage of the heat transfer fluid in tanks | n.a. | 2011 |
| MicroCSP Solar Cooling at Fort Bliss | USA | n.a. | PTC | 0.29 th | n.a. | Commercial | n.a. | n.a. | n.a. | n.a. | Xceltherm 600 (Thermal oil) | 98 | n.a. | n.a. | n.a. | n.a. | n.a. | n.a. | n.a. | 2011 |
| MicroCSP Solar Cooling at Masdar | Madagascar | n.a. | PTC | 0.13 | n.a. | Commercial | n.a. | n.a. | n.a. | n.a. | n.a. | n.a. | n.a. | n.a. | n.a. | n.a. | n.a. | n.a. | n.a. | 2011 |
| Palma de Rio 1 | Spain | Acciona Energia, Mistubihi Corp | PTC | 50 | 100 | Commercial | n.a. | n.a. | 0.27 | Rankine - Steam | Biphenyl/Diphenyl Oxide | 393 | n.a. | n.a. | Wet | 403000 | No storage | No storage | No storage | 2011 |
| Termesol 50 | Spain | NextEra | PTC | 50 | 170 | Commercial | Solar /Natural Gas (Evaporator) | 230 | n.a. | Rankine - Steam | Diphenyl/Biphenyl Oxide | 393 | 100 | n.a. | Wet | 510120 | Sensible | Molten salts (2 Indirect tanks) | 7,5 | 2011 |
| Archimede | Italia | ENEL | PTC | 5 | 9.2 | R&D | Solar/ Gas (combined cycle) | 8 | n.a. | n.a. | Solar salt (60% NaNO3, 40% KNO3) | 550 | 93 | Franco Tosi | Wet | 31860 | Sensible | Molten salts (2 direct tanks) | 8 | 2010 |
| BBEnergy Linear Fresnel Demo Plant | South Africa | BBEnergy | LFR | 0.08 | n.a. | R&D | n.a. | n.a. | n.a. | n.a. | n.a. | n.a. | n.a. | n.a. | n.a. | n.a. | n.a. | n.a. | n.a. | 2010 |
| Cameo  [4] | USA | Xcel Energy | PTC | 2 | n.a. | Commercial | Solar(1-2% de la production)/ Charbon | 2 | n.a. | Rankine - Steam | Xceltherm 600 (Thermal oil) | 300 | n.a. | n.a. | n.a. | 6640 | No storage | No storage | No storage | 2010 |
| Exresol-2 | Spain | Cobra, GE, KGAL | PTC | 50 | 170 | Commercial | Solar/Natural Gas(Evaporator HTF-15% max) | 200 | 0.27 | Rankine - Steam | Diphenyl/Diphenyl Oxide | 393 | 100 | Siemens SST700 | Wet (tower) | 510120 | Sensible | Molten salts (2 Indirect tanks) | 7,5 | 2010 |
| La Florida | Spain | Renovables SAMCA | PTC | 50 | 170 | Commercial | Solar/Natural Gas(Evaporator HTF-15% max) | 200 | 0.27 | Rankine - Steam | Diphenyl/Biphenyl Oxide | 393 | 100 | Siemens SST700 | Wet | 552750 | Sensible | Molten salts (2 Indirect tanks) | 7,5 | 2010 |
| Majadas I | Spain | Acciona Energia, Mistubihi Corp | PTC | 50 | 100 | Commercial | Solar/Natural Gas(Evaporator HTF-15% max) | n.a. | 0.27 | Rankine - Steam | Dowtherm A (Biphenyl / Diphenyl Oxide) | n.a. | n.a. | GE | Wet | n.a. | No storage | No storage | No storage | 2010 |
| Maricopa solar | USA | Tessera Solar | PTC | 1.5 | n.a. | Demonstration | No backup | n.a. | n.a. | n.a. | n.a. | n.a. | n.a. | n.a. | n.a. | n.a. | No storage | No storage | No storage | 2010 |
| Martin Next Generation Solar Energy Center | USA | Florida Power & Light | PTC | 75 | 155 | Commercial | Solar /Natural Gas (combined cycle) | 202 | n.a. | n.a. | Dowtherm A (Biphenyl / Diphenyl Oxide) | 348 | n.a. | n.a. | Wet | 464908 | No storage | No storage | No storage | 2010 |
| Palma de Rio 2 | Spain | Acciona Energia, Mistubihi Corp | PTC | 50 | 100 | Commercial | n.a. | n.a. | 0.27 | Rankine - Steam | Biphenyl/Diphenyl Oxide | 393 | n.a. | n.a. | Wet | 403000 | No storage | No storage | No storage | 2010 |
| Solar Beam Down Plant | United Arab Emirates | Cosmo Oil, Masdar, Tokyo Institute of technology | SPT | 0.1 | n.a. | R&D | n.a. | n.a. | n.a. | n.a. | n.a. | n.a. | n.a. | n.a. | n.a. | n.a. | n.a. | n.a. | n.a. | 2010 |
| Yazd integrated solar combined power plant | Iran | n.a. | PTC | 17 | n.a. | Commercial | Solar/Diesel | n.a. | n.a. | n.a. | Thermal oil | n.a. | n.a. | n.a. | n.a. | n.a. | n.a. | n.a. | n.a. | 2010 |
| Alvarado I (La Risca) | Spain | Acciona energia/Mitsubihi Corp | PTC | 50 | 100 | Commercial | Solar/Natural Gas(Evaporator HTF-15% max) | 135 | 0.27 | Rankine - Steam | Biphenyl/Diphenyl Oxide | 393 | 100 | Siemens SST700 | Wet | 352854 | No storage | No storage | No storage | 2009 |
| Andasol-2 | Spain | Antin Cobra RREEF | PTC | 50 | 170 | Commercial | Solar/Natural Gas(Evaporator HTF-15% max) | 200 | 0.27 | Rankine - Steam | Dowtherm A (Biphenyl / Diphenyl Oxide) | 393 | 100 | Siemens SST700 | Wet (tower) | 510000 | Sensible | Molten salts (2 direct tanks) | 7,5 | 2009 |
| AORA Solar Tulip Tower-Samar | Israel | AORA | SPT | 0.1 | n.a. | Demonstration | n.a. | n.a. | n.a. | n.a. | n.a. | n.a. | n.a. | n.a. | n.a. | n.a. | n.a. | n.a. | n.a. | 2009 |
| Eureka | Spain | Abengoa Solar | SPT | 2 | n.a. | R&D | n.a. | n.a. | n.a. | n.a. | Superheated steam | n.a. | n.a. | n.a. | n.a. | 4200 | Sensible | Steam | n.a. | 2009 |
| Exresol-1 | Spain | Cobra | PTC | 50 | 170 | Commercial | Solar/Natural Gas(Evaporator HTF-15% max) | 200 | 0.27 | Rankine - Steam | Dowtherm A (Biphenyl / Diphenyl Oxide) | 393 | 100 | Siemens SST700 | Wet (tower) | 510120 | Sensible | Molten salts (2 Indirect tanks) | 7,5 | 2009 |
| Holaniku at keahole Point | USA | Keahole Solar Power | PTC | 2 | 4 | Commercial | No backup | 1 | n.a. | Rankine - Steam | Xceltherm 600 (Thermal oil) | 176 | n.a. | n.a. | Wet | n.a. | Sensible | Réservoirs | 2 | 2009 |
| Ibersol Ciudad Real | Spain | IDAE, Iberdrola | PTC | 50 | 100 | Commercial | Solar/Natural Gas(Evaporator HTF-15% max) | 150 | 0.27 | Rankine - Steam | Biphenyl/Diphenyl Oxide | 391 | 100 | Siemens | Wet | 287000 | No storage | No storage | No storage | 2009 |
| KGDS Linear Fresnel CSP demonstration plant | India | KGDS Renewable Energy | LFR | n.a. | n.a. | Demonstration | No backup | n.a. | n.a. | n.a. | Water/Steam | 257 | n.a. | n.a. | n.a. | 1400 | No storage | No storage | No storage | 2009 |
| Planta Solar 20 | Spain | Abengoa Solar | SPT | 20 | n.a. | Commercial | Solar /Natural Gas (Evaporator) | 55 | 0.27 | Rankine - Steam | Water | 300 | n.a. | Franco Tosi - Dual Casing Turbine | Wet (tower) | 75216 | Sensible | Pressurized steam | 1 | 2009 |
| Puerto Errado 1 Thermosolar Power Plant | Spain | Novatec Solar | LFR | 1.4 | 2 | Commercial | n.a. | n.a. | 0.27 | n.a. | Water | 270 | n.a. | KKK - Siemens | Dry | n.a. | Sensible | 1 tank thermocline | 0,5 | 2009 |
| Sierra SunTower | USA | eSolar | SPT | 5 | n.a. | Demonstration | No backup | n.a. | n.a. | Rankine - Steam | Water | 440 | n.a. | n.a. | Wet | 27670 | No storage | No storage | No storage | 2009 |
| Solnova 3 | Spain | Abengoa Solar | PTC | 50 | 100 | Commercial | Solar /Natural Gas (Evaporator) | 110 | 0.27 | Rankine - Steam | Thermal oil | 393 | 100 | Siemens SST700 | Wet (tower) | 300000 | No storage | No storage | No storage | 2009 |
| Solnova 4 | Spain | Abengoa Solar | PTC | 50 | 100 | Commercial | Solar /Natural Gas (Evaporator) | 110 | 0.27 | Rankine - Steam | Thermal oil | 393 | 100 | Siemens SST700 | Wet (tower) | 300000 | No storage | No storage | No storage | 2009 |
| Andasol-1 | Spain | Antin Cobra RREEF | PTC | 50 | 170 | Commercial | Solar/Natural Gas(Evaporator HTF-12% max) | 200 | 0.27 | Rankine - Steam | Dowtherm A (Biphenyl / Diphenyl Oxide) | 393 | 100 | Siemens SST700 | Wet (tower) | 510000 | Sensible | Molten salts (2 direct tanks) | 7,5 | 2008 |
| BrightSource SEDC | Israel | BrightSource Energy | SPT | 6 | n.a. | Demonstration | n.a. | n.a. | n.a. | n.a. | n.a. | n.a. | n.a. | n.a. | n.a. | n.a. | n.a. | n.a. | n.a. | 2008 |
| Jülich Solar Tower | Germany | DLR | SPT | 1.5 | n.a. | R&D | No backup | 8 | n.a. | n.a. | Air | 680 | n.a. | Siemens | Dry | 17650 | Sensible | Ceramic | 1,5 | 2008 |
| Kimberlina Solar Thermal Power Power Plant | USA | Areva | LFR | 5 | n.a. | Demonstration | No backup | n.a. | n.a. | Rankine - Steam | Water | n.a. | 40 | n.a. | n.a. | 26000 | No storage | No storage | No storage | 2008 |
| Nevada Solar One | USA | Acciona Energia | PTC | 64 | 134 | Commercial | Solar/ Natural Gas (combined cycle) | 162 | n.a. | Rankine - Steam | Dowtherm A (Biphenyl / Diphenyl Oxide) | 393 | 86 | Siemens SST700 | n.a. | 357000 | Sensible | Salts | 0.5 | 2007 |
| Planta Solar 10 | Spain | Abengoa Solar | SPT | 11 | 24 | Commercial | Solar /Natural Gas (Evaporator) | 55 | 0.27 | Rankine - Steam | Water | 300 | n.a. | n.a. | Wet (tower) | 75216 | Sensible | Pressurized steam | 0,5 | 2006 |
| Saguato Power Plant | USA | Arizona Public Service | PTC | 1 | 2 | Commercial | No backup | 6 | n.a. | Rankine - Organic | Xceltherm 600 (Thermal oil) | 300 | 22 | Ormat | Wet | 100000 | No storage | No storage | No storage | 2006 |
| Shiraz CSP demonstration plant (Mehr-Niroo project) | Iran | n.a. | PTC | 0.25 | n.a. | Demonstration | n.a. | n.a. | n.a. | Rankine - Steam | Behran (Thermal Oil) | 265 | n.a. | n.a. | Wet | n.a. | n.a. | n.a. | n.a. | 2006 |
| CSIRO SolarGas | Australia | CSIRO | SPT | 0.5 | n.a. | n.a. | n.a. | n.a. | n.a. | n.a. | Water/Gas | n.a. | n.a. | n.a. | n.a. | n.a. | No storage | No storage | No storage | 2005 |
| ENEA SOLTERM | Italia | n.a. | PTC | n.a. | n.a. | R&D | n.a. | n.a. | n.a. | n.a. | n.a. | n.a. | n.a. | n.a. | n.a. | n.a. | n.a. | n.a. | n.a. | 2004 |
| Solgate | Spain | n.a. | n.a. | 0.3 | n.a. | n.a. | Solar/ Fossil | n.a. | n.a. | n.a. | Air | n.a. | n.a. | n.a. | n.a. | n.a. | n.a. | n.a. | n.a. | 2002 |
| TSA | Spain | n.a. | n.a. | 1 | n.a. | n.a. | n.a. | n.a. | n.a. | n.a. | Air | n.a. | n.a. | n.a. | n.a. | n.a. | Sensible | Ceramic | n.a. | 1993 |
| SEGS 9 | USA | NextEra | PTC | 80 | n.a. | Commercial | Solar /Natural Gas (Evaporator) | n.a. | n.a. | Rankine - Steam | Therminol (Synthetic oil) | 390 | 40 | MHI (Regenaration turbine) | n.a. | 483960 | No storage | No storage | No storage | 1990 |
| SEGS 4 | USA | NextEra | PTC | 30 | n.a. | Commercial | Solar /Natural Gas (Evaporator) | n.a. | n.a. | Rankine - Steam | Therminol (Synthetic oil) | 349 | 40 | MHI (Regenaration turbine) | n.a. | 230300 | No storage | No storage | No storage | 1989 |
| SEGS 5 | USA | NextEra | PTC | 30 | n.a. | Commercial | Solar /Natural Gas (Evaporator) | n.a. | n.a. | Rankine - Steam | Therminol (Synthetic oil) | 349 | 40 | MHI (Regenaration turbine) | n.a. | 250500 | No storage | No storage | No storage | 1989 |
| SEGS 6 | USA | NextEra | PTC | 30 | n.a. | Commercial | Solar /Natural Gas (Evaporator) | n.a. | n.a. | Rankine - Steam | Therminol (Synthetic oil) | 390 | 100 | MHI (Regenaration turbine) | n.a. | 188000 | No storage | No storage | No storage | 1989 |
| SEGS 7 | USA | NextEra | PTC | 30 | n.a. | Commercial | Solar /Natural Gas (Evaporator) | n.a. | n.a. | Rankine - Steam | Therminol (Synthetic oil) | 390 | 100 | MHI (Regenaration turbine) | n.a. | 194280 | No storage | No storage | No storage | 1989 |
| SEGS 8 | USA | NextEra | PTC | 80 | n.a. | Commercial | Solar /Natural Gas (Evaporator) | n.a. | n.a. | Rankine - Steam | Therminol (Synthetic oil) | 390 | 100 | MHI (Regenaration turbine) | n.a. | 464340 | No storage | No storage | No storage | 1989 |
| Weizmann Insttute of Science | Israel | n.a. | SPT | n.a. | n.a. | R&D | n.a. | n.a. | n.a. | n.a. | n.a. | n.a. | n.a. | n.a. | n.a. | n.a. | n.a. | n.a. | n.a. | 1988 |
| SPP-5 | Russia | n.a. | n.a. | 5 | n.a. | n.a. | n.a. | n.a. | n.a. | n.a. | Steam | n.a. | n.a. | n.a. | n.a. | n.a. | Sensible | Water/Steam | n.a. | 1986 |
| SEGS 2 | USA | Cogentrix | PTC | 30 | n.a. | Commercial | Solar /Natural Gas (Evaporator) | n.a. | n.a. | Rankine - Steam | Therminol (Synthetic oil) | 316 | 40 | MHI (Regenaration turbine) | n.a. | 190338 | No storage | No storage | No storage | 1985 |
| SEGS 3 | USA | NextEra | PTC | 30 | n.a. | Commercial | Solar /Natural Gas (Evaporator) | n.a. | n.a. | Rankine - Steam | Therminol (Synthetic oil) | 349 | 40 | MHI (Regenaration turbine) | n.a. | 230300 | n.a. | n.a. | n.a. | 1985 |
| MSEE/Cat B | USA | n.a. | n.a. | n.a. | n.a. | n.a. | n.a. | n.a. | n.a. | n.a. | Solar salt (60% NaNO3, 40% KNO3) | n.a. | n.a. | n.a. | n.a. | n.a. | Sensible | Solar salt (60% NaNO3, 40% KNO3) | n.a. | 1984 |
| SEGS I | USA | Cogentrix | PTC | 14 | n.a. | Commercial | No backup | n.a. | n.a. | Rankine - Steam | Therminol (Synthetic oil) | 307 | 40 | MHI (Regenaration turbine) | n.a. | 82960 | Sensible | Molten salts (2 direct tanks) | 3 | 1984 |
| THEMIS | France | n.a. | SPT | 2.5 | n.a. | R&D | n.a. | 102 | n.a. | n.a. | High technology salt | n.a. | n.a. | n.a. | n.a. | n.a. | Sensible | High technology salt | n.a. | 1984 |
| CESA 1 (PSA) | Spain | CIEMAT | SPT | 7 | n.a. | R&D | No backup | n.a. | n.a. | n.a. | n.a. | n.a. | n.a. | n.a. | n.a. | n.a. | Sensible | Molten salts | n.a. | 1983 |
| PSA CESA-1 | Spain | n.a. | n.a. | 1 | n.a. | n.a. | n.a. | n.a. | n.a. | n.a. | Steam | n.a. | n.a. | n.a. | n.a. | n.a. | n.a. | salts Nitrate | n.a. | 1983 |
| EURELIOS | Italia | n.a. | n.a. | 1 | n.a. | n.a. | n.a. | n.a. | n.a. | n.a. | Steam | n.a. | n.a. | n.a. | n.a. | n.a. | n.a. | salts Nitrate / Water | n.a. | 1981 |
| PSA SSPS-CRS | Spain | n.a. | SPT | 0.5 | n.a. | R&D | n.a. | n.a. | n.a. | n.a. | Liquid sodium | n.a. | n.a. | n.a. | n.a. | n.a. | n.a. | Sodium | n.a. | 1981 |
| SUNSHINE | Japan | n.a. | n.a. | 1 | n.a. | n.a. | n.a. | n.a. | n.a. | n.a. | Steam | n.a. | n.a. | n.a. | n.a. | n.a. | n.a. | salts Nitrate / Water | n.a. | 1981 |
