# Supplementary material for: Technical data for concentrated solar power plants in operation, under construction and in project
Source: Data Brief. 2017 Jun 23;13:597–9. doi: 10.1016/j.dib.2017.06.030 (PMC5499030; doi:10.1016/j.dib.2017.06.030)
Supplement: Supplementary file 3 — Supplementary material [file mmc3.docx]

**Table 2: Technical date for concentrated solar power plants under construction [1],[2]**

| **Project** | **Country** | **Owner** | **CSP Technology** | **Solar power (MWel)** | **Generation (GW.h /year)** | **Purpose of the plant** | **Hybridization** | **Area of the plant (hectare)** | **Electricity cost (€/kW.h)** | **Type of power cycle - fluid** | **Heat transfer fluid** | **Operating temperature (°C)** | **Operating pressure (bar)** | **Type of Turbin** | **Type of cooling** | **mirror's area (m^2)** | **Type of storage** | **Mean of storage** | **Storage capacity (h)** | **Start date** |
| --- | --- | --- | --- | --- | --- | --- | --- | --- | --- | --- | --- | --- | --- | --- | --- | --- | --- | --- | --- | --- |
| Abhijeet | India | Corporate Ispat Alloys | PTC | 50 | n.a. | Commercial | No backup | n.a. | 0.16 | Rankine - Steam | Therminol VP-1 (Biphenyl / Diphenyl Oxide) | n.a. | n.a. | Siemens SST700 | Wet | n.a. | No storage | No storage | No storage | n.a. |
| Agua Prieta II | Mexico | Comission Federal de Electricidad | PTC | 12 | 34 | Commercial | Solar / Gas | 60 | n.a. | Rankine - Steam | Thermal oil | n.a. | n.a. | n.a. | n.a. | 85000 | No storage | No storage | No storage | n.a. |
| Alba Nova 1  [5] | France | Solar Euromed | LFR | 12 | n.a. | Commercial | No backup | 23000 | n.a. | Rankine - Steam | Water | 300 | 65 | n.a. | Dry | 140000 | Other | Ruths tank | 1 | 2015 |
| Archetype SW550 | Italia | ENEL | PTC | 30 | n.a. | Demonstration | Solar / Biomass | n.a. | 0.21 | n.a. | n.a. | n.a. | n.a. | n.a. | n.a. | n.a. | Sensible | 2 tanks | n.a. | 2015 |
| Bokpoort | South Africa | ACWA Solafrica | PTC | 50 | 230 | Commercial | Solar/Fuel (LFO Boiler System (2x5 MWht)) | 100 | n.a. | Rankine - Steam | Dowtherm A (Biphenyl / Diphenyl Oxide) | 393 | n.a. | Siemens SST800 | Dry | 588660 | Sensible | Molten salts (2 indirect tanks) | 9.3 | 2015 |
| Cascades Rackam | Canada | Racjam | PTC | n.a. | n.a. | Commercial | Solar (reduction of gas consomation)/ Gas | n.a. | n.a. | n.a. | n.a. | n.a. | n.a. | n.a. | n.a. | 1500 | n.a. | n.a. | n.a. | n.a. |
| Centrale Solar Thermodynamique Llo [6] | France | CNIM | LFR | 9 | 17 | Commercial | No backup | 23 | n.a. | n.a. | Water | 285 | 70 | n.a. | Dry | 116000 | Sensible | Storage of steam in tanks | 1 | 2015 |
| CPI Golmud Solar Thermal Power Plant | China | China Power Investments Corporation | PTC | 100 | n.a. | Commercial | n.a. | n.a. | n.a. | n.a. | n.a. | n.a. | n.a. | n.a. | n.a. | n.a. | n.a. | n.a. | n.a. | n.a. |
| Crescent Dunes Solar Energy Project (Tonopah) | Nevada, USA | Solar Reserved | SPT | 110 | 500 | Commercial | No backup | n.a. | 1.08 | Rankine - Steam | Solar salt (60% NaNO3, 40% KNO3) | 565 | 115 | Alstom MT | n.a. | 1071161 | Sensible | Molten salts (2 direct tanks) | 10 | 2014 |
| Delingha Supcon Tower Plant | China | Zhejiang SUPCON Solar Energy Technology | SPT | 50 | 130 | Commercial | No backup | 330 | n.a. | n.a. | n.a. | n.a. | n.a. | n.a. | n.a. | 434880 | Sensible | Molten salts | n.a. | n.a. |
| Diwakar | India | Lanco Infratech | PTC | 100 | n.a. | Commercial | No backup | n.a. | 0.14 | Rankine - Steam | Synthetic oil | n.a. | n.a. | Siemens SST700 | n.a. | n.a. | Sensible | Molten salts (2 indirect tanks) | 4 | n.a. |
| Genesis Solar Energy Project | USA | NextEra | PTC | 250 | 300 | Commercial | Solar /Gas naturel (Evaporator) | 790 | n.a. | Rankine - Steam | Therminol VP-1 (Biphenyl / Diphenyl Oxide) | 393 | n.a. | n.a. | Dry | n.a. | 0 | 0 | 0 | n.a. |
| Gujarat | India | Aurum Renewable Energy | PTC | 20 | n.a. | Commercial | n.a. | n.a. | n.a. | n.a. | n.a. | n.a. | n.a. | n.a. | n.a. | n.a. | n.a. | n.a. | n.a. | n.a. |
| Gujarat Solar One | India | Cargo Power & Infrastructure | PTC | 25 | n.a. | Commercial | No backup | n.a. | n.a. | Rankine - Steam | Diphyl | 393 | n.a. | n.a. | Wet (tower) | 280000 | Sensible | Molten salts (2 indirect tanks) | 9 | n.a. |
| HelioFocus China Orion Project | China | TaiQing | PDC | 60 | n.a. | Commercial | n.a. | n.a. | n.a. | n.a. | Air | n.a. | n.a. | n.a. | n.a. | n.a. | n.a. | n.a. | n.a. | n.a. |
| Himin Solar Fresnel Demo Plant | China | Himin Solar | LFR | 2.5 | 5,25 | Demonstration | n.a. | n.a. | n.a. | n.a. | n.a. | n.a. | n.a. | n.a. | n.a. | 32000 | n.a. | n.a. | n.a. | n.a. |
| Huaneng Sanya | China | China Huaneng Group | LFR | 1.5 | n.a. | Demonstration | n.a. | n.a. | n.a. | n.a. | n.a. | n.a. | n.a. | n.a. | n.a. | n.a. | n.a. | n.a. | n.a. | n.a. |
| KaXu Solar One | South Africa | Abengoa | PTC | 100 | 320 | Commercial | No backup | n.a. | 0.26 | Rankine - Steam | Dowtherm A (Biphenyl / Diphenyl Oxide) | n.a. | 100 | n.a. | Dry | 800000 | Sensible | Molten salts (2 indirect tanks) | 2.5 | n.a. |
| Khi Solar One | South Africa | Abengoa | SPT | 50 | 190 | Commercial | No backup | n.a. | n.a. | Rankine - Steam | Water/Steam | n.a. | n.a. | n.a. | Dry | 580000 | Sensible | Saturated steam | 3 | n.a. |
| Kogan Creek Solar Boost | Australie | CS Energy | LFR | 44 | 0,44 | Commercial | No backup | n.a. | n.a. | Rankine - Steam | Water/Steam | 370 | 60 | Siemens | Dry | n.a. | No storage | No storage | No storage | n.a. |
| KVK Energy Solar Project | India | KVK Energy, Lanco Solar Energy | PTC | 100 | n.a. | Commercial | No backup | n.a. | n.a. | n.a. | Thermal oil | n.a. | n.a. | Siemens SST700 | Wet | n.a. | Sensible | Molten salts (2 indirect tanks) | 4 | n.a. |
| Megha Solar Plant | India | Megha Ingeneering & infrastructure | PTC | 50 | n.a. | Commercial | n.a. | n.a. | 0.15 | Rankine - Steam | Thermal oil | n.a. | n.a. | GE | n.a. | n.a. | Sensible | Molten salts (2 indirect tanks) | 8 | n.a. |
| MicroCSP Process Heat at Frabelle | Papua New Guinea | n.a. | PTC | 1 th | n.a. | Commercial | n.a. | n.a. | n.a. | n.a. | Water | 97 | n.a. | n.a. | n.a. | n.a. | n.a. | n.a. | n.a. | n.a. |
| MicroCSP Solar Cooling at Davis-Monthan Air Force Base | USA | n.a. | PTC | 0.18 th | n.a. | Commercial | n.a. | n.a. | n.a. | n.a. | Water | 176 | n.a. | n.a. | n.a. | n.a. | n.a. | n.a. | n.a. | n.a. |
| MicroCSP Solar Cooling at Holcim | Mexico | n.a. | PTC | 0.29 th | n.a. | Commercial | n.a. | n.a. | n.a. | n.a. | Water | 95 | n.a. | n.a. | n.a. | n.a. | n.a. | n.a. | n.a. | n.a. |
| Mojave Solar Project | USA | Abengoa Solar | PTC | 250 | 600 | Commercial | Solar /Gas naturel (Evaporator) | 714 | n.a. | Rankine - Steam | Therminol VP-1 (Biphenyl / Diphenyl Oxide) | n.a. | n.a. | GE A-14 | Wet | n.a. | No storage | No storage | No storage | n.a. |
| Ningxia ISCC | China | Hanas New Energy Group | PTC | 92 | n.a. | Commercial | Oui | n.a. | n.a. | n.a. | n.a. | n.a. | n.a. | n.a. | n.a. | n.a. | Oui | n.a. | n.a. | n.a. |
| NOOR 1 | Morocco | ACWA, Aries, MASEN, TSK | PTC | 160 | n.a. | Commercial | n.a. | n.a. | 0.15 | n.a. | Thermal oil | n.a. | n.a. | n.a. | n.a. | n.a. | Sensible | Molten salts (2 indirect tanks) | 3 | n.a. |
| Planta Solar Cerro Dominador | Chile | Abengoa | SPT | 110 | n.a. | Commercial | n.a. | 1400 | n.a. | Rankine - Steam | Molten salts (Sodium and potassium nitrates) | 550 | n.a. | n.a. | Dry | n.a. | Sensible | Molten salts (2 direct tanks) | 20 | 2018 |
| Projeto Helioterm | Brasil | Centro de Pesquisas de Energia Elecrica | PTC | 1 | n.a. | n.a. | n.a. | n.a. | n.a. | n.a. | n.a. | n.a. | n.a. | n.a. | n.a. | n.a. | n.a. | n.a. | n.a. | n.a. |
| Rajasthan Sun Technique - Dhursar | India | Reliance Power | LFR | 100 | n.a. | Commercial | n.a. | n.a. | n.a. | n.a. | n.a. | n.a. | n.a. | n.a. | Wet (tower) | n.a. | n.a. | n.a. | n.a. | n.a. |
| Supcon Solar Project | China | SUPCON Solar | SPT | 50 | n.a. | Commercial | n.a. | 330 | n.a. | Rankine - Steam | Molten salts | n.a. | n.a. | n.a. | n.a. | 434880 | Sensible | Molten salts (2 direct tanks) | 2.5 | n.a. |
| Tianwei 1.5 MW CSP pilot plant | China | China Datang Power Group | PTC | 1.5 | n.a. | R&D | n.a. | n.a. | n.a. | n.a. | n.a. | n.a. | n.a. | n.a. | n.a. | n.a. | n.a. | n.a. | n.a. | n.a. |
| Tooele Army Depot | USA | - | PDC | 1.5 | n.a. | Commercial | n.a. | n.a. | n.a. | Stirling | Helium | n.a. | n.a. | n.a. | Closed loop system | n.a. | No storage | No storage | No storage | n.a. |
| Yumen Gansu Solar Thermal Pilot Plant | China | Tianwei New Energy Holding, China Datang Group | PDC | 10 | n.a. | Demonstration | n.a. | n.a. | n.a. | n.a. | n.a. | n.a. | n.a. | n.a. | n.a. | n.a. | n.a. | n.a. | n.a. | n.a. |
