# Supplementary material for: Technical data for concentrated solar power plants in operation, under construction and in project
Source: Data Brief. 2017 Jun 23;13:597–9. doi: 10.1016/j.dib.2017.06.030 (PMC5499030; doi:10.1016/j.dib.2017.06.030)
Supplement: Supplementary file 4 — Supplementary material [file mmc4.docx]

**Table 3: Technical data for concentrated solar power plant planned [1],[2]**

| **Project** | **Country** | **Owner** | **CSP Technology** | **Solar power (MWel)** | **Generation (GW.h /year)** | **Purpose of the plant** | **Hybridization** | **Area of the plant (hectare)** | **Electricity cost (€/kW.h)** | **Type of power cycle - fluid** | **Heat transfer fluid** | **Operating temperature (°C)** | **Operating pressure (bar)** | **Type of Turbin** | **Type of cooling** | **mirror's area (m^2)** | **Type of storage** | **Mean of storage** | **Storage capacity (h)** | **State of the project** | **Construction start** |
| --- | --- | --- | --- | --- | --- | --- | --- | --- | --- | --- | --- | --- | --- | --- | --- | --- | --- | --- | --- | --- | --- |
| !Xun and Khwe solar power facility | South Africa | n.a. | LFR | 100 | n.a. | Commercial | No backup | n.a. | n.a. | n.a. | n.a. | n.a. | n.a. | n.a. | n.a. | n.a. | n.a. | n.a. | n.a. | Planned | n.a. |
| Abengoa Solar Jordan CSP plant | Jordan | Abengoa | n.a. | 25 | n.a. | Commercial | No backup | n.a. | n.a. | n.a. | n.a. | n.a. | n.a. | n.a. | n.a. | n.a. | n.a. | n.a. | n.a. | Planned | n.a. |
| Al Abdaliyah Integrated Solar Combined Cycle [7], [9] | Kuwait | Kuwait | n.a. | 60 | n.a. | Commercial | Solar /Gas | n.a. | 0.062 | n.a. | n.a. | n.a. | n.a. | n.a. | n.a. | n.a. | No storage | No storage | No storage | In developpement | n.a. |
| Ashalim CSP plant 1 | Israel | Alstom/Bright source energy | SPT | 121 | n.a. | Commercial | n.a. | n.a. | 0.16 | n.a. | n.a. | n.a. | n.a. | n.a. | n.a. | n.a. | n.a. | n.a. | n.a. | In developpement | 2017 |
| Ashalim CSP plant 2 | Israel | AbengoaShikun & Binui Renewable Energy | PTC | 121 | n.a. | Commercial | Solar/PV/Natural gas | n.a. | 0.16 | Rankine - Steam | Therminol VP-1 (Biphenyl / Diphenyl Oxide) | n.a. | n.a. | n.a. | n.a. | n.a. | Sensible | Molten salts (2 indirect tanks) | 4.5 | In developpement | 2017 |
| Bhadla Solar Park | India | n.a. | n.a. | 100 | n.a. | Commercial | n.a. | n.a. | n.a. | n.a. | n.a. | n.a. | n.a. | n.a. | n.a. | n.a. | n.a. | n.a. | n.a. | In developpement | 2015 |
| BrighSource Coyote springs 1 [8] | Nevada, USA | BrightSource Energy | SPT | 200 | 573 | Commercial | n.a. | n.a. | n.a. | Rankine - Steam | Water | n.a. | n.a. | n.a. | n.a. | n.a. | n.a. | n.a. | n.a. | In developpement | n.a. |
| BrighSource Coyote springs 2 | Nevada, USA | BrightSource Energy | SPT | 200 | 573 | Commercial | n.a. | n.a. | n.a. | Rankine - Steam | Water | n.a. | n.a. | n.a. | n.a. | n.a. | n.a. | n.a. | n.a. | In developpement | n.a. |
| BrighSource PG&E 5 | California, USA | BrightSource Energy | SPT | 200 | 573 | Commercial | n.a. | n.a. | n.a. | Rankine - Steam | Water | n.a. | n.a. | n.a. | n.a. | n.a. | n.a. | n.a. | n.a. | In developpement | n.a. |
| BrighSource PG&E 6 | California, USA | BrightSource Energy | SPT | 200 | 573 | Commercial | n.a. | n.a. | n.a. | Rankine - Steam | Water | n.a. | n.a. | n.a. | n.a. | n.a. | n.a. | n.a. | n.a. | In developpement | n.a. |
| BrighSource PG&E 7 | California, USA | BrightSource Energy | SPT | 200 | 573 | Commercial | n.a. | n.a. | n.a. | Rankine - Steam | Water | n.a. | n.a. | n.a. | n.a. | n.a. | n.a. | n.a. | n.a. | In developpement | n.a. |
| Campu Giiavesu | Italia | Campu Giiavesu | PTC | 30 | 129 | Commercial | No backup | n.a. | - | Rankine - Steam | Molten salts (Sodium and potassium nitrates) | - | - | - | Dry | - | Sensible | Molten salts (2 indirect tanks) | 15 | Planned | n.a. |
| Catalyst Private Equity Jordan CSP plant | Jordan | n.a. | n.a. | 50 | n.a. | Commercial | n.a. | n.a. | n.a. | n.a. | n.a. | n.a. | n.a. | n.a. | n.a. | n.a. | n.a. | n.a. | n.a. | n.a. | n.a. |
| Chile CSP plant | Chile | n.a. | n.a. | 10 | n.a. | Demonstration | Solar / Fuel (max 6%) | n.a. | n.a. | n.a. | n.a. | n.a. | n.a. | n.a. | n.a. | n.a. | n.a. | n.a. | 3 | In developpement | n.a. |
| Collinsville Hybrid CSP-gas project | Australia | RATCH Australia | LFR | 30 | n.a. | Commercial | Solar(integrated in a vapor cycle)/ Gas | n.a. | n.a. | n.a. | n.a. | n.a. | n.a. | n.a. | n.a. | n.a. | n.a. | n.a. | n.a. | Planned | n.a. |
| Coremas | Brasil | Abantia Braxenergy | PTC | 50 | n.a. | Commercial | n.a. | n.a. | n.a. | n.a. | n.a. | n.a. | n.a. | n.a. | n.a. | n.a. | n.a. | n.a. | n.a. | Planned | n.a. |
| Crossroads Solar Energy Project | USA | SolarReserve | SPT | 150 | n.a. | Commercial | n.a. | 1036 | n.a. | n.a. | Molten salts (Sodium and potassium nitrates) | n.a. | n.a. | n.a. | n.a. | n.a. | n.a. | n.a. | n.a. | Planned | n.a. |
| Delingha Solar Thermal Plant | China | China Guangdong Nuclear Power Group | PTC | 50 | 199 | Commercial | No backup | n.a. | n.a. | n.a. | n.a. | n.a. | n.a. | n.a. | n.a. | n.a. | n.a. | n.a. | 7 | In developpement | n.a. |
| DLR - Algeria CSP tower pilot plant | Algeria | DLR | SPT | 7 | n.a. | Demonstration | n.a. | n.a. | n.a. | n.a. | n.a. | n.a. | n.a. | n.a. | n.a. | n.a. | n.a. | n.a. | n.a. | In developpement | n.a. |
| eCare Solar Thermal Project | Morocco | CNIM | LFR | 1 | 1.6 | Demonstration | No backup | n.a. | n.a. | Rankine - Organic | Water | 280 | 70 | n.a. | Dry | 10000 | Sensible | Storage of steam in tanks | 2 | En Dévolloptment | n.a. |
| EJRE Maan CSP plant | Jordan | EJRE | n.a. | 50 | n.a. | Commercial | n.a. | n.a. | n.a. | n.a. | n.a. | n.a. | n.a. | n.a. | n.a. | n.a. | n.a. | n.a. | n.a. | Planned | n.a. |
| El Borma ISCC | Tunesia | SITEP STEG | SPT | 5 | n.a. | Commercial | No backup | n.a. | n.a. | n.a. | n.a. | n.a. | n.a. | n.a. | n.a. | 40000 | 0 | 0 | 0 | Planned | 2013 |
| EOS Cyprus | Cyprus | Alfa Mediterranean Enterprises | SPT | 25 | n.a. | Commercial | n.a. | n.a. | 0.26 | n.a. | n.a. | n.a. | n.a. | n.a. | n.a. | n.a. | Sensible | Graphit blocs | n.a. | Planned | 2012 |
| Eskom CSP plant | South Africa | n.a. | SPT | 100 | n.a. | Commercial | No backup | n.a. | n.a. | Rankine - Steam | n.a. | n.a. | n.a. | n.a. | n.a. | n.a. | Sensible | Molten salts (2 direct tanks) | n.a. | Planned | n.a. |
| Evolution Solar Jordan CSP plant | Jordan | n.a. | n.a. | 50 | n.a. | Commercial | n.a. | n.a. | n.a. | n.a. | n.a. | n.a. | n.a. | n.a. | n.a. | n.a. | n.a. | n.a. | n.a. | Planned | n.a. |
| Flumini Mannu | Italia | Flumini Mannu | PTC | 50 | 215 | Commercial | Solar/ Diesel (booster) | 269 | n.a. | Rankine - Steam | Molten salts (Sodium and potassium nitrates) | n.a. | n.a. | n.a. | Dry | 815600 | Sensible | Molten salts (2 direct tanks) | 15 | Planned | n.a. |
| Gaskell Sun Tower | USA | eSolar | SPT | 245 | n.a. | Commercial | n.a. | 445 | n.a. | Rankine - Steam | Water | n.a. | n.a. | n.a. | n.a. | n.a. | n.a. | n.a. | n.a. | Planned | n.a. |
| Giave Bonorva | Italia | Sunwise Capital | PTC | 50 | 215 | Commercial | No backup | 235 | n.a. | Rankine - Steam | n.a. | n.a. | n.a. | n.a. | Dry | n.a. | Sensible | Molten salts (2 direct tanks) | n.a. | Planned | n.a. |
| Gonnosfanadiga | Italia | Gonnosfanadiga | PTC | 50 | 215 | Commercial | Solar/ Diesel (booster) | 224 | n.a. | Rankine - Steam | Molten salts (Sodium and potassium nitrates) | n.a. | n.a. | n.a. | Dry | 779332 | Sensible | Molten salts (2 direct tanks) | 15 | Planned | n.a. |
| HelioFocus Ramat Hovav | Israel | n.a. | PDC | 1 | n.a. | Commercial | n.a. | n.a. | n.a. | n.a. | n.a. | n.a. | n.a. | n.a. | n.a. | n.a. | n.a. | n.a. | n.a. | Planned | n.a. |
| Helios Power | Cyprus | n.a. | PDC | 50.76 | n.a. | Commercial | n.a. | 200 | n.a. | n.a. | n.a. | n.a. | n.a. | n.a. | n.a. | n.a. | n.a. | n.a. | n.a. | In developpement | n.a. |
| Hidden Hills SEGS | USA | BrightSource Energy | SPT | 500 | n.a. | Commercial | n.a. | 1327 | n.a. | Rankine - Steam | n.a. | n.a. | n.a. | n.a. | Dry | n.a. | n.a. | n.a. | n.a. | In developpement | n.a. |
| Huadian Jinta Solar Thermal Power Plant | China | China Huadian | PTC | 50 | n.a. | Commercial | n.a. | n.a. | n.a. | n.a. | n.a. | n.a. | n.a. | n.a. | n.a. | n.a. | n.a. | n.a. | n.a. | Planned | n.a. |
| Huludao CSP plant | China | Huludao Ruixinda Industry | PTC | 50 | n.a. | Commercial | n.a. | n.a. | n.a. | n.a. | n.a. | n.a. | n.a. | n.a. | n.a. | n.a. | n.a. | n.a. | n.a. | Planned | n.a. |
| Hyder Valley Solar Energy Project | USA | Hyberdrola | PTC | 235 | n.a. | n.a. | n.a. | n.a. | n.a. | n.a. | n.a. | n.a. | n.a. | n.a. | n.a. | n.a. | n.a. | n.a. | n.a. | Planned | 2014 |
| Ilanga CSP 1 | South Africa | Ilangathu Solar Power | PTC | 100 | n.a. | Commercial | n.a. | n.a. | n.a. | Rankine - Steam | n.a. | n.a. | n.a. | n.a. | n.a. | n.a. | Sensible | Molten salts (2 indirect tanks) | 4.5 | In developpement | 2014 |
| India One Solar Thermal Power Plant | India | n.a. | PDC | 1 | n.a. | Demonstration | n.a. | n.a. | n.a. | n.a. | n.a. | n.a. | n.a. | n.a. | n.a. | n.a. | n.a. | n.a. | n.a. | Planned | n.a. |
| Kom Ombo CSP project | Egypt | NREA | PTC | 100 | n.a. | Commercial | n.a. | n.a. | n.a. | n.a. | n.a. | n.a. | n.a. | n.a. | n.a. | n.a. | n.a. | n.a. | n.a. | In developpement | n.a. |
| Lentini | Italia | n.a. | PTC | 50 | n.a. | Commercial | Solar/ Natural gas | 100 | n.a. | Rankine - Steam | n.a. | n.a. | n.a. | n.a. | Dry | n.a. | Sensible | Molten salts (2 direct tanks) | n.a. | Planned | n.a. |
| Maximus Dish project | Greece | n.a. | PDC | 75 | n.a. | Commercial | n.a. | n.a. | n.a. | n.a. | n.a. | n.a. | n.a. | n.a. | n.a. | n.a. | n.a. | n.a. | n.a. | In developpement | n.a. |
| Mazara Solar | Italia | n.a. | SPT | 50 | n.a. | Commercial | n.a. | n.a. | n.a. | n.a. | Steam | n.a. | n.a. | n.a. | n.a. | n.a. | Sensible | Steam | n.a. | In developpement | n.a. |
| Mejillones | Chile | GDF Suez, Solar Power Group | LFR | 5 | n.a. | Commercial | n.a. | n.a. | n.a. | n.a. | n.a. | n.a. | n.a. | n.a. | n.a. | n.a. | n.a. | n.a. | n.a. | Planned | n.a. |
| Metsimatala CLFR Power Plant | South Africa | n.a. | LFR | 30 | n.a. | Commercial | n.a. | n.a. | n.a. | n.a. | n.a. | n.a. | n.a. | n.a. | n.a. | n.a. | n.a. | n.a. | n.a. | Planned | n.a. |
| MINOS CSP tower | Greece | n.a. | SPT | 50 | n.a. | Demonstration | n.a. | 143 | n.a. | n.a. | Steam | n.a. | n.a. | n.a. | n.a. | n.a. | No storage | No storage | No storage | In developpement | n.a. |
| Mitsubishi Jordan CSP plant | Jordan | Mitsubihi Corp | n.a. | 50 | n.a. | Commercial | n.a. | n.a. | n.a. | n.a. | n.a. | n.a. | n.a. | n.a. | n.a. | n.a. | n.a. | n.a. | n.a. | Planned | n.a. |
| North Midlands Solar Thermal Power Project | Australia | Carbon Reduction Ventury, Solastor | SPT | 3 | n.a. | Commercial | n.a. | n.a. | n.a. | n.a. | n.a. | n.a. | n.a. | n.a. | n.a. | n.a. | n.a. | n.a. | n.a. | Planned | n.a. |
| Ordos Solar Thermal Power Plant | China | China Datang Corporation | SPT | 50 | 120 | Demonstration | Solar/Natural gas(Evaporator HTF-10% max) | n.a. | 0.12 | n.a. | n.a. | n.a. | n.a. | n.a. | n.a. | n.a. | n.a. | n.a. | 5 | In developpement | n.a. |
| Ouarzazate 2 | Morocco | MASEN | SPT | 100 | n.a. | Commercial | n.a. | n.a. | n.a. | n.a. | n.a. | n.a. | n.a. | n.a. | n.a. | n.a. | oui | n.a. | n.a. | In developpement | n.a. |
| Ouarzazate 3 | Morocco | MASEN | SPT | 200 | n.a. | Commercial | n.a. | n.a. | n.a. | n.a. | n.a. | n.a. | n.a. | n.a. | n.a. | n.a. | oui | n.a. | 3 | In developpement | n.a. |
| Palen SEGS | USA | BrightSource Energy | SPT | 500 | 1412 | Commercial | Solar /Natural gas (max 2%) | n.a. | n.a. | Rankine - Steam | Steam | n.a. | n.a. | n.a. | Dry | n.a. | No storage | No storage | No storage | In developpement | 2016 |
| Palmdale Hybrid Power Plant | USA | n.a. | PTC | 50 | n.a. | Commercial | n.a. | 152 | n.a. | n.a. | Therminol VP-1 (Biphenyl / Diphenyl Oxide) | n.a. | n.a. | n.a. | n.a. | n.a. | No storage | No storage | No storage | Planned | n.a. |
| Pedro de Valdivia | Chile | Ibereolica | PTC | 360 | n.a. | Commercial | Solar/ Natural gas | 1982 | n.a. | n.a. | Thermal oil | 393 | n.a. | n.a. | Dry | n.a. | Sensible | Molten salts (2 indirect tanks) | 10.5 | In developpement | n.a. |
| Planta Termosolar Maria Elena | Chile | Ibereolica | SPT | 400 | 2589 | Commercial | n.a. | 2897 | n.a. | n.a. | Molten salts (Sodium and potassium nitrates) | 565 | n.a. | n.a. | Dry | n.a. | Sensible | Molten salts (2 direct tanks) | n.a. | Planned | n.a. |
| Planta Termosolar Pedro de Valdivia | Chile | Ibereolica | SPT | 360 | 2108 | Commercial | n.a. | n.a. | n.a. | n.a. | n.a. | n.a. | n.a. | n.a. | n.a. | n.a. | n.a. | n.a. | n.a. | In developpement | n.a. |
| PTC50 Alvarado | Spain | Acciona Energia | SPT | 50 | n.a. | Demonstration | Solar/ Biomass | n.a. | n.a. | n.a. | n.a. | n.a. | n.a. | n.a. | n.a. | n.a. | n.a. | n.a. | n.a. | In developpement | n.a. |
| Quartzsite | USA | SolarReserve | SPT | 100 | 500 | Commercial | n.a. | n.a. | n.a. | Rankine - Steam | Molten salts (Sodium and potassium nitrates) | n.a. | n.a. | n.a. | Dry | n.a. | n.a. | n.a. | n.a. | Planned | n.a. |
| Rajasthan Solar One | India | Entegra limited | PTC | 10 | n.a. | Commercial | n.a. | n.a. | n.a. | n.a. | n.a. | n.a. | n.a. | n.a. | n.a. | n.a. | n.a. | n.a. | n.a. | Planned | n.a. |
| Rice Solar Energy Project (RSEP) | California, USA | SolarReserve | SPT | 150 | 448 | Commercial | No backup | 600 | n.a. | Rankine - Steam | Molten salts | - | 115 | - | Dry | 1071361 | Sensible | Molten salts (2 direct tanks) | 10 | In developpement | n.a. |
| Saguache Solar Energy Project | USA | SolarReserve | SPT | 200 | 900 | Commercial | n.a. | n.a. | n.a. | n.a. | n.a. | n.a. | n.a. | n.a. | n.a. | n.a. | n.a. | n.a. | n.a. | Planned | n.a. |
| Salta CSP plant | Argnetina | Harbine Turbine Company, Solar Noa | PTC | 20 | n.a. | Commercial | n.a. | n.a. | n.a. | n.a. | n.a. | n.a. | n.a. | n.a. | n.a. | n.a. | n.a. | n.a. | n.a. | Planned | n.a. |
| Shagaya project KISR | Kuwait | Kuwait Institute for Scientific Research | PTC | 50 | n.a. | Demonstration | oui | n.a. | n.a. | n.a. | n.a. | n.a. | n.a. | n.a. | Dry | n.a. | Sensible | Molten salts (2 indirect tanks) | n.a. | Planned | n.a. |
| Shneur Solar Thermal Power Plant | Israel | n.a. | PTC | 120 | n.a. | Commercial | n.a. | n.a. | n.a. | n.a. | n.a. | n.a. | n.a. | n.a. | n.a. | n.a. | n.a. | n.a. | n.a. | Planned | n.a. |
| Solastor Mejillones | Chile | Safe Earth Energy, Solastor | STP | 5 | n.a. | Commercial | n.a. | n.a. | n.a. | n.a. | n.a. | n.a. | n.a. | n.a. | n.a. | n.a. | Sensible | Graphit blocs | n.a. | Planned | n.a. |
| Sundt Solar Boost | USA | Tuscon Electricity Power | LFR | 5 | n.a. | Commercial | n.a. | n.a. | n.a. | n.a. | n.a. | n.a. | n.a. | n.a. | n.a. | n.a. | n.a. | n.a. | n.a. | In developpement | n.a. |
| TAQA Concentrated Solar Power Plant | Egypt | TAQA Arabia | SPT | 250 | n.a. | Commercial | n.a. | n.a. | n.a. | n.a. | n.a. | n.a. | n.a. | n.a. | n.a. | n.a. | n.a. | n.a. | n.a. | Planned | n.a. |
| Tibet Solar Thermal Power Plant | China | China Huaneng Group | PTC | 50 | n.a. | Commercial | n.a. | n.a. | n.a. | n.a. | n.a. | n.a. | n.a. | n.a. | n.a. | n.a. | n.a. | n.a. | n.a. | Planned | n.a. |
| TN-STEG Concentrated Solar Power plant | Tunesia | STEG | PTC | 50 | n.a. | Commercial | Solar/ Natural gas (Evaporator HTF-15% max) | n.a. | n.a. | n.a. | n.a. | n.a. | n.a. | n.a. | n.a. | n.a. | No storage | No storage | No storage | Planned | n.a. |
| TuNur | Tunesia | Glory Clean Energy, Nur Energie, TOP Oilfield Services | SPT | 2000 | n.a. | Commercial | n.a. | n.a. | n.a. | n.a. | Molten salts | 550 | n.a. | n.a. | n.a. | n.a. | Sensible | Molten salts (2 direct tanks) | n.a. | In developpement | n.a. |
| Two Sigma CSP plant | Israel | Two Sigma | PTC | 60 | n.a. | Commercial | n.a. | n.a. | n.a. | n.a. | n.a. | n.a. | n.a. | n.a. | n.a. | n.a. | n.a. | n.a. | n.a. | Planned | n.a. |
| Victorville 2 Hybrid Power Plant | USA | City of Victorville | PTC | 50 | n.a. | Commercial | n.a. | 100 | n.a. | n.a. | n.a. | n.a. | n.a. | n.a. | n.a. | n.a. | No storage | No storage | No storage | Planned | n.a. |
| Whyalla Solar Oasis | Australia | NP Power, Sustainable partners, Wizard Power | PDC | 40 | n.a. | Commercial | n.a. | n.a. | n.a. | n.a. | n.a. | n.a. | n.a. | n.a. | n.a. | n.a. | n.a. | n.a. | n.a. | Planned | n.a. |
| Xina Solar One | South Africa | Abengoa | PTC | 100 | n.a. | Commercial | n.a. | n.a. | n.a. | n.a. | n.a. | n.a. | n.a. | n.a. | n.a. | n.a. | Sensible | Molten salts (2 indirect tanks) | 5 | In developpement | n.a. |
| Zeenni Trading Agency CSP plant Bsarma El Koura | Lebanon | Zeeni Trading Agency | PTC | 2.8 | n.a. | Commercial | n.a. | n.a. | n.a. | n.a. | Solar salt (60% NaNO3, 40% KNO3) | n.a. | n.a. | n.a. | n.a. | n.a. | Sensible | Molten salts (2 direct tanks) | n.a. | Planned | n.a. |
| Zeenni's Trading Agency 50 MW CSP plant | Lebanon | Zeeni Trading Agency | PTC | 50 | n.a. | Commercial | n.a. | n.a. | n.a. | n.a. | n.a. | n.a. | n.a. | n.a. | n.a. | n.a. | n.a. | n.a. | n.a. | Planned | n.a. |

**References**

[1]*,* [2]*,* [1,2][3], [4], [5], [6], [7], [8], [9]

[1] CSP World. CSP World Map 2015. http://www.csp-world.com/cspworldmap.

[2] Global Energy Observatory 2012. http://globalenergyobservatory.org/.

[3] Airlight Energy. AIT-BAHA CSP Pilot Plant 2015. http://www.airlightenergy.com/ait-baha-pilot-plant.

[4] Scott C. World’s first hybrid coal-solar power plant goes online in colorado 2010. http://inhabitat.com/worlds-first-hybrid-coal-solar-power-plant-goes-online-in-colorado/.

[5] Solar EUROMED. Alba Nova 1 2017. http://www.solareuromed.com/alba-nova-1.

[6] Division Solaire CNIM. CENTRALE SOLAIRE THERMODYNAMIQUE Llo. 2013.

[7] Meed Insight. Mena Solar Power Market and Projects Report 2014. 2014.

[8] Energy Division. Pubilic utilities commission of the state of california 2009. http://docs.cpuc.ca.gov/PUBLISHED/FINAL_RESOLUTION/107761.htm.

[9] Koweït Ministry of Electricity and water. Al Abdaliyah Integrated Solar Combined Cycle (ISCC) 2015.

http://www.ptb.gov.kw/en/Al-Abdaliyah-Integrated-Solar-Combined-Cycle-(ISCC).
